# Recent Advances in Molecular Mechanisms of the NKG2D Pathway in Hepatocellular Carcinoma

**DOI:** 10.3390/biom10020301

**Published:** 2020-02-14

**Authors:** Jian Wang, Cun-Di Li, Lin Sun

**Affiliations:** Department of Aetiology and Immunology, Medical College, Anhui University of Science & Technology, Huainan 232001, Anhui, China

**Keywords:** NKG2D, ligand, signal transduction, cytokines, perforin, hepatocellular carcinoma, oncolytic effect

## Abstract

Hepatocellular carcinoma is a common malignant tumor with high mortality. Its malignant proliferation, invasion, and metastasis are closely related to the cellular immune function of the patients. NKG2D is a key activated and type II membrane protein molecule expressed on the surface of almost all NK cells. The human NKG2D gene is 270 kb long, located at 12p12.3–p13.1, and contains 10 exons and 9 introns. The three-dimensional structure of the NKG2D monomeric protein contains two alpha-helices, two beta-lamellae, and four disulfide bonds, and its’ signal of activation is transmitted mainly by the adaptor protein (DAP). NKG2D ligands, including MICA, MICB, and ULBPs, can be widely expressed in hepatoma cells. After a combination of NKG2D and DAP10 in the form of homologous two polymers, the YxxM motif in the cytoplasm is phosphorylated and then signaling pathways are also gradually activated, such as PI3K, PLCγ2, JNK-cJunN, and others. Activated NK cells can enhance the sensitivity to hepatoma cells and specifically dissolve by releasing a variety of cytokines (TNF-α and IFN-γ), perforin, and high expression of FasL, CD16, and TRAIL. NK cells may specifically bind to the over-expressed MICA, MICB, and ULBPs of hepatocellular carcinoma cells through the surface activating receptor NKG2D, which can help to accurately identify hepatoma, play a critical role in anti-hepatoma via the pathway of cytotoxic effects, and obviously delay the poor progress of hepatocellular carcinoma.

## 1. Introduction

Hepatocellular carcinoma (HCC) is one of the most common malignancies in humans originating from liver epithelial cells and has a high recurrence rate [1,2]. It has high mortality and global distribution being the fifth most common cancer and the second main reason of cancer-related mortality [3]. It has been confirmed that the incidence of HCC is high in Asia, Africa, and southern Europe [4]. The occurrence of hepatocellular carcinoma is a complex pathological process mediated by a variety of undesirable factors and accumulated over a long period [5]. Chronic alcoholism [6], nitrosamine [7], and aflatoxin [8] can induce liver cancer. In particular, cirrhosis of the liver after HBV infection can easily develop to liver cancer without active treatment [9]. The malignant proliferation, invasion, and metastasis of HCC cells have been associated with the NK cell immune function of the patients [10], such as the cytotoxicity of primary NK cells from the hepatoma patients against SMMC7721 or HepG2 cells displayed a significant reduction, and also were positively associated with the level of miR-506 and negatively correlated with the mRNA level of STAT3 [11].

NKG2D (Natural Killer group 2 member D), a key member of the C type lectin receptor family, a lectin-like type 2 transmembrane glycoprotein, is an important activating receptor widely expressed on the surface of NK cells, CD8+αβT cells, γδ T cells, and NK T cells [12]. Some cytokines, such as IL-2, IL-15, IFN-γ, etc., can significantly up-regulate the level of NKG2D on the surface of NK cells [13]. High expression of NKG2D in NK cells can further promote the activation of NK cells [14]. NKG2D ligands include MHC class I chain related molecule (MIC) and MHC-I related molecules UL16 binding protein (ULBP). Under normal physiological conditions, NKG2D ligands are usually at a poor or absent expression in most normal cells, such as gastrointestinal epithelial cells and endothelial cells. It is worth noting that NKG2D ligands are widely expressed in virus-infected cells, liver cancer, colon cancer, lung cancer, and other common tumors. NKG2D binds to the corresponding ligands with different affinities ranging from 6 to 9000 nM and activates downstream cascade signaling, which plays an important role in the antiviral and anti-tumor immune response [15]. Therefore, the response of NK cells to acute stimulation is dynamically regulated by the interaction of NKG2D on the surface of NK cells and related ligands of cell in the local microenvironment [16].

NKG2D regarded as the best-characterized activating receptor on NK cells, and the potentialities of hepatic infiltrating NK cells to play antitumor functions have been unclear. Its molecular mechanism of interaction between NKG2D and ligands and in the process of the immune response to hepatocellular carcinoma will be expressly focused in this review.

## 2. The Discovery of NKG2D

NKG2D, as a characteristic and novel receptor, was found on the surface of activated NK cells in the 90s of the last centuries. The DNA sequence of the single NKG2-D isolate was determined by recloning the PCR amplification fragment in both orientations into M13mp19 and applying the single-stranded sub-cloning method. During the DNA sequence analysis of NKG2 group on templates derived from the original l-Gem cDNA clones by the method of asymmetric PCR in 1991, Houchins and others [17] luckily found the dominant expression gene NKG2D, and also cloned and screened its adjacent NKG2A, NKG2B, and NKG2C at the same time. These groups of genes encode type II membrane proteins composed of 215–233 amino acids. The 5′ end of NKG2-D was 95% homologous with the stippled region within the transcripts of NKG2A, NKG2B, and NKG2C. Surprisingly, NKG2C and NKG2A had 94% homology in the external (COOH-terminal) domain and 56% homology in the transmembrane and intramembrane regions. However, there was only 21% amino acid homology between NKG2D and the other three group genes. The peptide sequence homology results confirmed that the NKG2 peptide is a member of a common supergene family with several type II membrane proteins.

**Figure 1:**
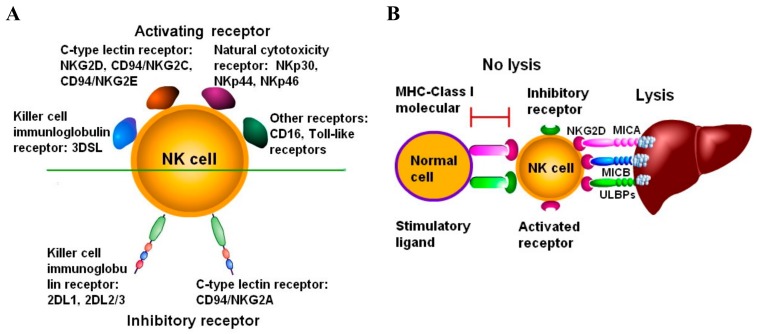
NK cell receptors and expression. (**A**) The types of receptors expressed on the surface of NK cells. Activating receptors can be expressed on NK cells, such as NKG2D, C, NKp46, CD16, and others, and multiple inhibitory receptors also can be expressed on NK cells, such as NKD2A, NKG2B, and so on. (**B**) Balanced expression of activating receptor and inhibitory receptor. Based on the dominant expression of inhibitory receptor, NK cells have no cytotoxic effect on normal cells with poor expression of NKG2D ligands.

NK cells recognize target cells based on NKG2D and play a crucial role in the anti-tumor immune response. By comparing with T cells and B cells, NKG2D expressed on the surface of NK cells is not restricted by MHC in the activation process. Through the adhering receptor, NKG2D recognized specifically the major histocompatibility complex class I polypeptide-related molecule A (MIC-A), molecule B (MIC-B), and MHC-I related molecules UL16 binding proteins (ULBPs) [18,19] on the surface of multiple tumor cells, which subsequently plays a pivotal cytotoxic effect on the tumor cells. A variety of receptors expressed on NK cells can be divided into active receptors and inhibitory receptors. The active receptors belong to the characteristic natural cytotoxicity receptor (NCR) family, such as NKp46, NKp30, NKp44 [20,21], and C type lectin family receptor (NKG2D, CD94/NKG2C, CD94/NKG2E, CD94/NKG2F, and CD161) [22], and also include active KIRs (killer immunoglobulin receptors), such as KIR2DS1, KIR2DS4, and KIR2DL4 [23]. The inhibitory receptors mainly include NKG2A, KIR2DL1, KIR2DL2/L3, and KIR3DL1 [24,25]. The characteristics of the activating receptor and inhibitory receptor on the surface of NK cells are shown in Table 1 and Figure 1.

The human NKG2 receptor family includes at least seven members, such as NKG2A, B, C, D, E, F, and H [27]. Among them, NKG2A and B belong to the member of inhibitory receptors. NKG2C, D, and E belong to the member of activating receptors. The expression of NKD2A, B, C, E, and H receptors on the surface of NK cells requires CD94 molecular to participate in assistance and to form disulfide-linked heterodimers with invariant CD94 molecular [28]. It is noteworthy that the cytoplasmic domain of human NKG2A contains two crucial immunoreceptor tyrosine-based inhibitory motifs (ITIMs), which can interact with SHP-1 (Src homology region 2 domain-containing phosphatase-1) and SHP-2 (Src homology region 2 domain-containing phosphatase-2) respectively [29]. These inhibitory motifs are composed of the specific sequence of I/VXYXXV/L in the cytoplasmic domain, which is conducive to further tyrosine phosphorylation of ITIMs after binding of receptor ligands [30]. SHP-1 tyrosine phosphatase can be recruited to dephosphorylate tyrosine residues, and which can activate cascade signaling molecules and transmit inhibitory signals. NKG2A mediated SHIP-1 signaling pathway plays a key role in mediating the occurrence and progression of hepatocellular carcinoma and other malignant tumors [31]. The overexpression of SHIP-1 can inhibit the proliferation, migration, and invasion of hepatoma cells. Conversely, knockdown of SHP-1 can significantly enhance the malignant proliferation of hepatocellular carcinoma.

Due to the absence of the ITIMs motif, MKG2C can be blocked to inhibit signal transmission and can transmit the activated signal in the form of disulfide-linked heterodimers with the invariant CD94 molecular. A lot of LAK cells (lymphokine-activated killer cells) derived from C57BL/6 were successfully prepared from C57BL/6J, BALB/cJ, and C57BL/6T murine in the experimental studies of Ho *et al.* [32]. By comparing the amino acid sequences of human NKG2D and human CD94, the protein sequence was deduced from the expressed sequence tag (EST) database, and one clone with high homologous to human NKG2D and two homologous clones from human CD94 were identified by the probes with the EST clone inserts. Two positive clones with 2.1 and 1.1.3 were screened from the cDNA library of C57BL/6J LAK cells by the EST probe hybridization to murine NKG2D (mNKG2D) and murine CD94 (mCD94). The positive cDNA clone with a 2.1 cell line encodes a type II integral membrane protein in homology to human NKG2D and murine NKG2D (NKR-P2) was 60% and 81%, respectively. However, the amino acid homology of type II integral membrane protein encoded by a positive cDNA clone in the 1.1.3 cell line in human NKG2D and NKR-P2 was 77% and 55%, respectively.

## 3. Chromosome Localization of NKG2D

### 3.1. Chromosome Localization of NKG2D in the Mouse

**Figure 2:**
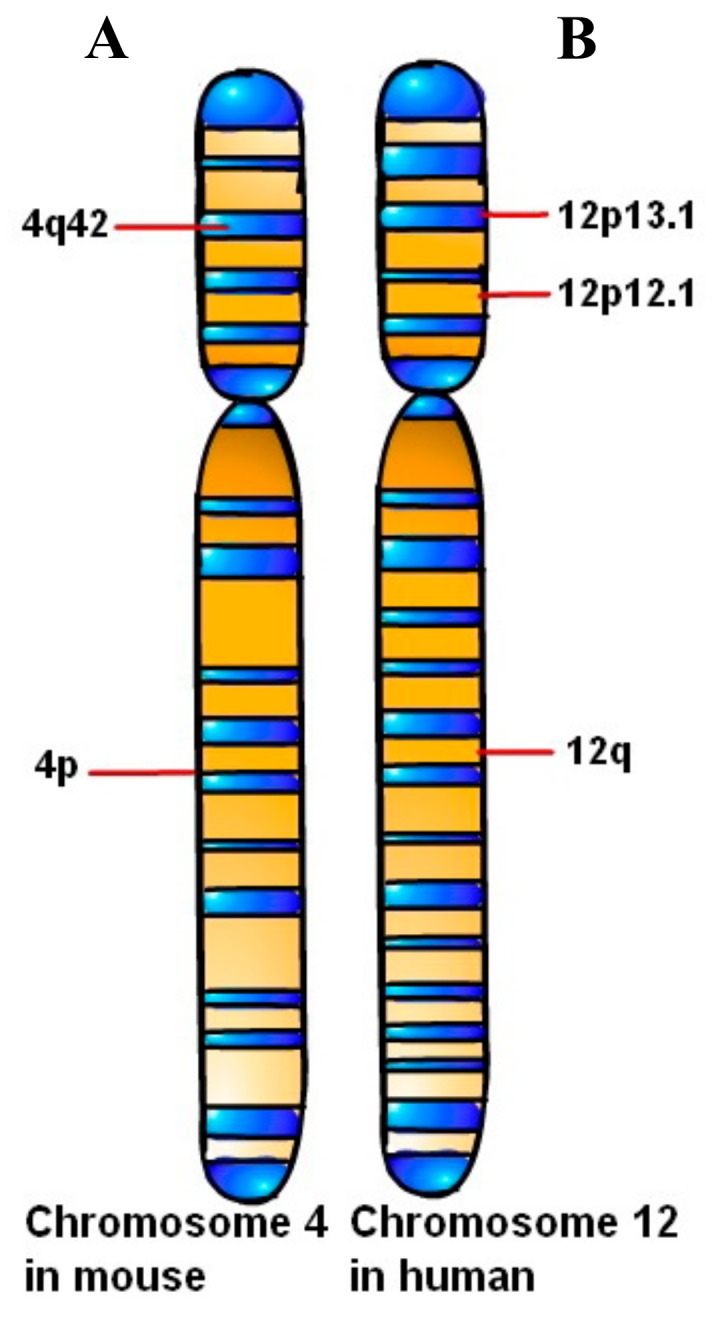
Chromosome localization of NKG2D. A: Mouse-derived NKG2D location on chromosome 4 and B: human-derived NKG2D location on chromosome 12.

As early as 1996, Dissen et al. [33] had verified that the genetic locus of the NK-mediated alloreactivity (NKa) induced by NK cells were located on the autosomes. It was an important dominant gene in the natural killer gene complex (NKC) region of the mouse, which could regulate the antimicrobial activity of allogeneic lymphocytes and NK cells. The NKC on the mouse chromosome 4 was identified (Figure 2A) by linkage analysis and pulsed-field gel electrophoresis, which could include the NK cell receptor protein 1 (NKR-P1) and Ly-49 multigene families plus the NKG2D homologue of the mouse.

### 3.2. Chromosome Localization of NKG2D in Human

The human *KLRK1* gene is located in 12p12.3-p13.1 of the chromosome with its genome size of approximately 270 kb, and encodes type II membrane protein with a molecular weight of 42 KDa [34]. In 2012, Imai et al. [35] had found that the NKG2D expressed on the surface of NK cells and CD8^+^T cells in the peripheral blood of 732 cases of atomic-bomb survivors were analyzed by the method of single nucleotide polymorphisms (SNPs), and confirmed that NKG2D in human located on chromosome 12p (Figure 2B). These SNPs had formed two crucial haplotypes such as NKG2D hb-1 and NKG2D hb-2. It was noteworthy that the low-activity-related LNK1 and high-activity-related HNK1 as two major haplotype alleles had been caused by NKG2D hb-1. Both haplotypes significantly correlated with the natural cytotoxic activity of NK cells and CD8^+^ T lymphocytes. HNK1/HNK1 haplotype could be more meaningful than LNK1/LNK1 in revealing cancer risk reduction. A case-control study based on the cohort study had demonstrated that the risk of cancer was significantly decreased in patients with expression of HNK1/HNK1 haplotypes than those of LNK1/LNK1. The mean fluorescence intensity (MFI) of NKG2D expression on NK and CD8^+^ T cells and NKG2D haplotypes significantly increased in the order of LNK1/LNK1, LNK1/HNK1 and HNK1/HNK1 (*P* = 0.003), which was consistent with the increasing order of natural cytotoxic activity. The individual haplotype markers of haplotype-tagging single nucleotide polymorphisms (htSNPs) showed that the main homozygote, heterozygote, and small homozygous genotype were in sequence (*P* = 0.02–0.003), and had obvious individual differences in the cytotoxic reaction to tumor cells in vivo.

### 3.3. The Difference Expression of NKG2D between Humans and Mice

**Figure 3:**
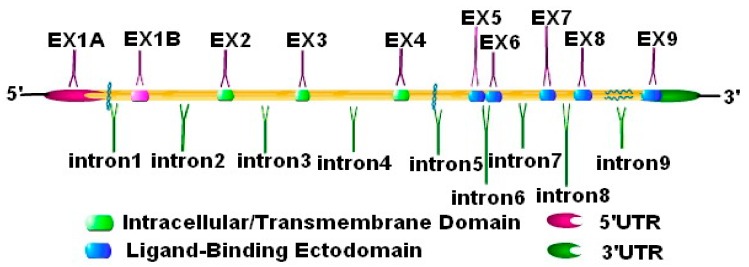
The gene structure of human NKG2D. The gene structure of human NKG2D is composed of 10 exons and 9 introns. Exons 2–4 encode the intracellular and transmembrane domain. Exons 5–8 encode the domain, which is prominent in the extracellular and can combine with a ligand.

NKG2D, also known as killer cell lectin-like receptor K1 (KLRK1), has a functional single nucleotide polymorphism (SNPs) [36], which has a gene length of 37793 bases and is located at 10416219–10454012 bp of the negative chain of chromosome 12. The data from the ENTREZ database show that it contains 12 exons. A synonymous and nonsynonymous substitution at two nucleotide positions have been identified in three alleles of human KLRK1, which may be greatly limited polymorphism. The limited polymorphism of homologous gene KLRK1 also has been confirmed in mice. KLRK1 orthologous genes existed in all genomes of mammalian and marsupial and are highly conserved during evolution (Figure 3). As an active receptor, NKG2D can be expressed in NK cells, CD8^+^T cells, and other immunoreactive cells in humans and mice, but the expression in CD4^+^T cells is usually absent [37]. The differential expression of NKG2D in humans and mice is shown as shown in Table 2.

## 4. Interaction between NKG2D and Ligands

**Figure 4:**
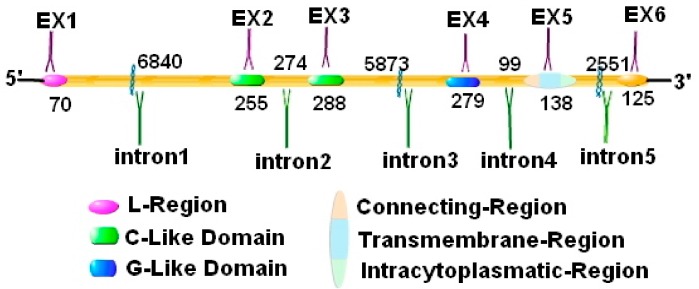
The gene structure of MICA. The gene structure of MICA contains 5 introns and 6 exons. Among them, exon 1 encodes the prepeptide, exons 2–4 encode three extracellular globular domains, exon 5 encodes the transmembrane domain, and exon 6 encodes the cytoplasmic tail structure.

MICA, MICB, and ULBPs are currently recognized as NKG2D specific ligands [47]. Take MICA as an example, its genetic structure is shown in Figure 4. NKG2D binds specifically to the different ligands with different affinity [48]. The affinity of NKG2D to corresponding ligands in humans is ULBP1, MICA, and MICB from high to low [49], but the affinity of ULBP2 to ULBP4 is still unclear. However, the affinity of NKG2D to its ligands in the mouse is RAE1δ, RAE1α, RAE1γ, RAE1β, H60, and MULT1 from high to low, but the affinity between NKG2D and its ligand RAE1ε has not been proved [50]. Surprisingly, the NKG2D of one species can bind to the NKG2D ligands of other species [51], such as mouse-derived NKG2D can also bind to the human-derived ULBP1 and ULBP2. Moreover, human-derived NKG2D also can be combined with the pig-derived NKG2D ligands.

Under the co-culture of NK cells and tumor cells that can express NKG2D ligands, the silent NK cells only show low levels of anti-tumor effects, which have suggested that the cytotoxicity of NK mediated by NKG2D may further require the participation of other signals. The hepatic liver infiltrating NK cells in tissues of human primary liver cancer and colorectal cancer (CRC) liver metastases were compared from the data of Easom et al. [52], their results showed that NK cells with high expression of CXCR6^+^CD69^+^ and NKG2D were widely expressed in tissues of HCC and liver colorectal metastases. However, the phenotype of NK cells was different in local hepatocellular carcinoma and the uninvolved distant liver margins. IL-15 could directly promote the anti-tumor activity of NK cells against HCC cell lines and hepatoma cells directly extracted from liver cancer tissues in vitro. The activation characteristics of CD8^+^/CD4^+^ T cells and NK cells induced by IL-15 made full use in studies of Hu et al. [53] and a novel molecule (called P22339) based on IL-15 was ingeniously constructed through a rational structure-based design. This study found that it could significantly inhibit the growth and metastasis of tumor in the rodent model both in vitro and in vivo, and also could activate the T and NK cells of the cynomolgus monkey, which showed great potential for cancer immunotherapy.

**Figure 5:**
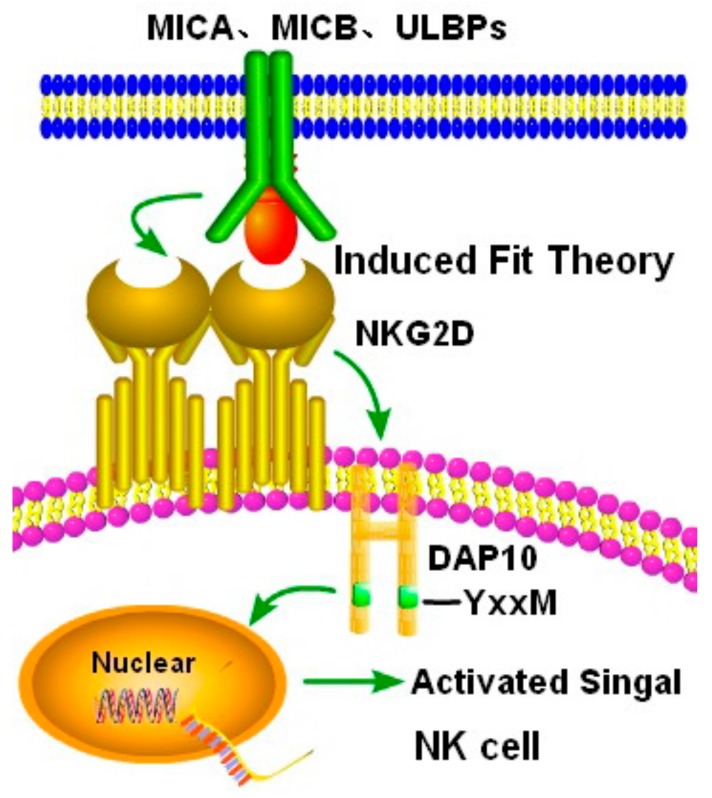
The interaction between NKG2D and its ligands. When NKG2D binds to its ligand, the transmembrane charged amino acid residues can be linked to TM residues of DAP10, and induce the phosphorylation of the YxxM motif in the cytoplasm, and then activate the downstream phosphoinositide-3 kinase (PI3K) signal pathway.

As a crucial activation receptor, NKG2D can specifically bind to DAP10 via the induced fit theory and further induce phosphorylation [54]. The meaning of induced fit theory is shown that when two molecules recognize and combine with each other, molecular flexibility plays a key role in the induced-fit effect to complete self-assembly [55]. DAP10, a kind of DNAX-associated protein 10, is related to the YxxM motif. The charged amino acid residues in the transmembrane (TM) region of the NKG2D homologous dimer are linked to the TM residues of one single DAP10 via the two salt bridges so as to form a hexamer structure (Figure 5). The direct measurement of chemometrics shows that a NKG2D homologous dimer is tightly linked to four chains of DAP10. Selective mutations in one of the base TM residues in NKG2D can lead to an absence of two chains in DAP10, which also strongly suggesting that arginine in each TM sequence can be used as a site for the interaction of DAP10 dimers. This mutation can significantly reduce the possibility of the formation of NKG2D dimer, which is harmful to the formation of the hexamer structure [56]. The binding of NKG2D to a single DAP10 ligand can lead to phosphorylation of its four chains, which may be related to the sensitivity of the NKG2D receptor signal transduction, especially in the case of a low level of DAP10. DAP10 can form a homologous dimer with disulfide bonds [57], and also with a key YxxM motif in the cytoplasm, which can bind to the p85 is the regulatory subunit of phosphoinositide-3 kinase (PI3K) after phosphorylation, so as to activate the signal transduction pathway of PI3K [58].

It has been also reported that the YxxM motif may be defined as the DAP10 functional region with a SH2 (Src homology 2 protein) structure, a highly conserved area, which has a TxxM motif similar to CD28 fragments in cytoplasm, and also can recruit 1,4,5- three phosphoric inositol (inositol-1,4,5-triphosphosate, IP3), and further bind to P85 subunits of phosphatidylinositide 3-kinase (PI-3K), and activate the downstream growth factor receptor-bound protein 2 (Grb2). The Grb2 molecule can synchronously combine with the protein of Shc and Sos so as to form the Shc-Grb2-Sos complex and then activate Sos. The activated Sos proteins bind with the Ras protein on the plasma membrane and further activate the Ras protein, so that the cascade reaction of the downstream signal is triggered [59]. After NKG2D binding to the ligands, the combination of Grb2 with the Vav of guanine nucleotide exchange factor (GEF) can make tyrosine phosphorylated at the amino terminus of the SLP76 (Src homology 2 domain containing leukocyte protein of 76000) protein. Tyrosine after phosphorylation can be combined with the SH2 domain of Vav1 to form a complex consisting of a variety of adapter proteins, and then activate the JNK kinase and play a pivotal cytotoxic activity. It can also release granulase through the signal pathway of phospholipase C gamma 2 (PLCã2) and exert multiple cytotoxicities [60].

SLP76 with a molecular weight of about 76 kD is a tyrosine phosphine protein with an SH2 domain [61]. Its amino terminal contains a PEST (Proline-Glutamate-Serine-Threonine rich) domain and several tyrosine residues, which as an important tyrosine phosphorylation sites. Its central region contains a large number of proline domains, while carboxyl terminus contains a SH2 domain [62]. The characteristics of the proline rich domain and multiple phosphorylation sites in SLP76 proteins are easy to directly bind to the SH3 domain of the other joint protein Grb2 to form a complex consisting of a variety of adapter proteins, which can be able to transmit extracellular signals to the Ras protein. The activated Ras is further combined with the amino terminal of serine/threonine protein kinase Raf1, which can phosphorylate extracellular signal regulated kinase (ERK), and eventually lead to oxidative stress and activation of transcription factors such as NF-kappa B, and also triggers a series of potent cytotoxic effects.

## 5. Activation of NKG2D Promotes the Activity of NK Cells Against Hepatocellular Carcinoma

**Figure 6:**
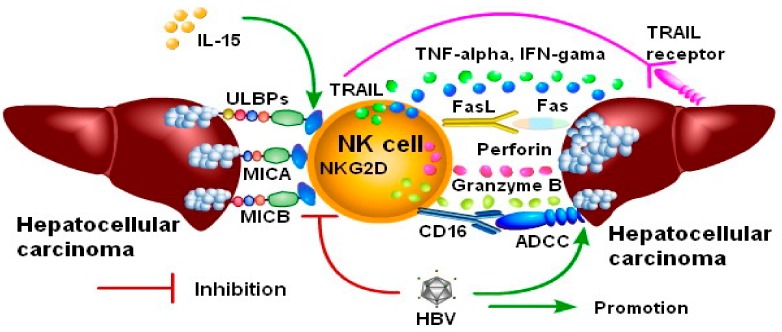
NKG2D on activated NK cells play crucial inhibitory effects against hepatocellular carcinoma. The activated NK cells can express NKG2D, release multiple cytokines, secrete granzyme, express FasL, TRAIL, promote the target cells dissolve, and play effective anti-hepatoma effects in many ways. Both HBV particles and soluble ligand sMICA produced by hepatoma cells can effectively inhibit NK cell activation.

Hepatocellular carcinoma (HCC) as a common malignant tumor has some significant characteristics of expansive growth, exogenic growth, and invasive growth, which makes it easy to infiltrate the surrounding tissue space, blood vessels, and lymphatic vessels, and also actively penetrate the capillary walls into the bloodstream and metastasize along with the blood vessels [63] and lymphatic channels [64]. In addition, hepatocellular carcinoma can also be spread by seeding [65]. The activation of NKG2D receptors can promote the release of multiple active proteins from NK cells, such as perforin and granzymes, and can also induce the expression of tumor necrosis factor (TNF) ligand interferon-gamma (IFN-γ) to induce apoptosis of hepatocarcinoma and is beneficial to clean up the tumor (Figure 6). An exciting novel research results also had been shown that the NK cells with positive CD56^bright^ located in human hepatic sinuses had effective cytotoxicity against SNU398 hepatoma cells through multiple signaling pathways (such as high expression in NKG2D, NKp46, TRAIL, FasL, and others), which were very helpful for the immunotherapy of hepatocellular carcinoma [66].

### 5.1. TNF-alpha Secreted by NK Cells Exerts Anti-hepatocarcinoma Activity by Activated NKG2D

TNF-alpha (TNF-α) is a small molecule and secretory glycoprotein produced by activated macrophages, NK cells, and so on. It not only widely participates in the immune defense and inflammatory response of cell activation [67], survival, proliferation, necrosis, and apoptosis, but also plays multiple antitumor effects via the way of inducement of tumor cells swollen and lysis [68]. Radiofrequency ablation (RFA) is an effective and low risk treatment for hepatocellular carcinoma, and is especially suitable for patients with liver cancer, which are not suitable for operation. A total of five New Zealand white rabbits aged between two to three months, weighing 2.5–3.0 kg were randomly selected and established as an animal model of hepatocellular carcinoma in the novel studies of Mo et al. [69]. Two weeks later, the expression level of NKG2D receptors on NK cells in the white rabbits were all detected by flow cytometry and the levels of IFN-γ and TNF-α were tested by ELISA on basis of hematoxylin and eosin (H&E) staining of the liver tissue on the slices. The exciting results showed that the number of NK cells in the rabbit tumor model decreased significantly (*P <* 0.01). After the treatment of RFA, the number of NK cells and the expression of NKG2D receptors increased significantly and reached the peak value at the end of the 1st week after the treatment of RFA. The levels of IFN-gamma and TNF-alpha also increased significantly during the same period, and the peak value appeared at the first weekend and lasted until the fourth week under the treatment of RFA. These results had been suggested that RFA could significantly enhance the immunotherapeutic effects of NK cells on hepatocellular carcinoma through the up-regulation of NKG2D expression.

The regulation role of NKG2D receptors on NK cells in hepatocellular carcinoma and precancerous lesions was analyzed by Zekri et al. [70]. The levels of the active NK cells (CD56^+^CD161^+^), the activated NK cells (CD56^+^CD314^+^), and the inactive NK cells (CD56^+^CD158^+^) from the peripheral blood of the patients with liver cancer, liver cirrhosis, and chronic hepatitis were regarded as the major detection indices and comparatively analyzed in detail. The results showed that NKG2D expression was significantly down-regulated in NK cells, and without NKG2D expression was found in nearly 63% of HCC cases. The secretion of IL-2, IFN-alpha, and IFN-gamma also decreased significantly, but the levels of TNF-alpha-R2 (soluble tumor necrosis factor receptor type II), IL-10, and IL-1 beta increased significantly in HCC patients. It had been suggested that NKG2D could up-regulate the expression of TNF-alpha-R2 and promote the secretion of TNF-alpha to inhibit the malignant proliferation of hepatocellular carcinoma cells. Inhibition of TNF-alpha expression can promote the recurrence and metastasis of hepatocellular carcinoma. Xu et al. [71]. found that long-term use of Indomethacin could induce the expression of PD-1 and PD-L2, and inhibit the secretion of TNF-alpha and IFN-gamma through TRIF/NF-kappa B axis and JAK/STAT3 axis in a dose-dependent manner *in vivo* and *in vitro*, and further promote intrahepatic recurrence and extrahepatic distant metastasis of hepatocellular carcinoma. When the expression of PD-1 and PD-L2 was blocked, the decrease of TNF-alpha and IFN-gamma induced by Indomethacin could be easily reversed. It was suggested that Indomethacin should be used cautiously when cancer pain occurred in patients with hepatocellular carcinoma to prevent recurrence and malignant metastasis of hepatocellular carcinoma caused by the down-regulation of TNF-alpha and IFN-gamma triggered by the over-use of Indomethacin.

### 5.2. IFN-gamma Secreted by NK Cells Exerts Anti-hepatocarcinoma Activity by Activated NKG2D

In order to clarify that NKG2D could promote the secretion of IFN and play an inhibitory role in hepatocellular carcinoma cells, the detailed experimental research data on Wu et al. [72] had been found that the natural killer cell dysfunction induced by monocytes/macrophages in local hepatocellular carcinoma tissues was mediated by the interaction of CD48/2B4. On the basis of comparative observation of NK cell infiltration and accumulation in a human normal liver (distal normal tissues of hepatic hemangioma), liver of chronic hepatitis (liver transplantation), non-tumorous liver, and paired intratumoral tissues, the survival of patients was predicted. The low level accumulation and infiltration of NK cells and less secretion of TNF-α and IFN-γ were discovered in 294 cases with untreated and advanced hepatocellular carcinoma. There was a positive correlation between the NK cells dysfunction and the high infiltration of monocytes/macrophages in the local peritumoral stroma. The intense expression of the CD48 protein in monocytes was a key factor to induce NK cell dysfunction in cancer focus. It had been suggested that the progression and immune escape of hepatocellular carcinoma were closely related to the decrease of the NK cell number and function (TNF-α and IFN-γ) in the tumor microenvironment. Lasfar et al. [73] took BNL mouse as a hepatoma model and found that IFN-alpha (IFN-α) could induce NK cell activation, significantly up-regulate the expression of NKG2D, and increase the secretion of IFN-gamma (IFN-γ). IFN-lambda (IFN-λ) could sensitize the hepatocytes of BNL hepatoma mice, and the combination of IFN-α and IFN-lambda could significantly enhance the targeted lysis of NK cells to hepatoma cells. The mortality of BNL hepatoma cells was decreased significantly after the application of anti-NKG2D monoclonal antibody, which suggested that the expression of NKG2D receptors on NK cells blocked by a monoclonal antibody might inhibit the secretion of IFN-γ and decrease the cytotoxicity effect on BNL hepatoma cells. However, the results by using flow cytometry staining to detect 30 HCC patients from Zhang et al. [74] had shown that there were a large number of CD11b-CD27-(DN) NK cell subsets infiltrating in the focal tissues of hepatocellular carcinoma. The subsets of CD11b-CD27-(DN) NK cells with low cytotoxicity and deficient IFN-gamma resulted in the dysfunction of NK cells in patients with HCC, which was positively correlated with the malignancy degree and size of tumors, and negatively correlated with the survival period of the patients. It was suggested that the poor prognosis of the patients with hepatocellular carcinoma could be positively associated with CD11b-CD27-(DN) NK cells infiltrating with insufficient secretion of IFN-gamma secretion in the focal tissues.

Recently, Xu et al. [75] have explored the role of pro-inflammatory response as another novel perspective in the malignant progression of tumors and the tumor immune escape. Under the inducement of frankincense and myrrh (FM), the effects of CD8^+^NKG2D^+^ T cells enriched from human peripheral blood were observed on hepatoma cell lines HCKM3 and Hepa 1-6. It was found that FM could significantly inhibit the signal transduction of NF-kappa B and STAT3 in HCC cells and further inhibit the activation of CD8^+^NKG2D^+^ T cells at a dose of 60 mg/kg. In HCC-bearing mice, FM at non-toxic doses (0.5 mg/mL) could not inhibit the tumor cells growth in immune-damaged mice and could significantly inhibit the growth of tumor cells in immunocompetent mice. The prolongation of the life span in HCC tumor bearing mice was based on the secretion of a large amount of IFN-gamma in the tumor microenvironment (TME) in immune competent mice. When the neutralizing antibodies were injected intraperitoneally to deplete CD8^+^ T cells or NK cells, the cytotoxicity to hepatocellular carcinoma was significantly weakened. On this basis, Hwang et al. [66] further explored the phenotype and function of NK cells in human hepatic sinusoids and their cytotoxicity to hepatocellular carcinoma cells. It was found that NKG2D, NKP46, TNF-related apoptosis-inducing ligand (TRAIL), and Fas ligand (FASL) were highly expressed in hepatic intrasinusoidal (HI) CD56^bright^ NK cells, which had little degranulation effect on Huh7 cells, but could secrete more IFN-γ to produce strong cytotoxicity on Huh7 cells. Interestingly, few significant changes in cytotoxicity of HI CD56^bright^ NK cells could be observed after a blockade of PD-L1. It had been suggested that HI CD56^bright^ NK cells might be the most effective functional cells for immunotherapy of hepatocellular carcinoma.

### 5.3. FasL Expressed on NK Cells Exerts Anti-hepatocarcinoma Activity by Activated NKG2D

Factor associated suicide (Fas) belongs to type I transmembrane glycoproteins with a molecular weight of 36 kDa, which can widely express a variety of virus-infected cells and tumor cells. Meanwhile, factor associated suicide ligand (FasL) is a type II transmembrane glycoprotein with a molecular weight of about 36–43 kDa, and is mainly expressed in activated T cells and NK cells [76]. As the interconnection between Fas and its ligand FasL, the cytotoxicity of tumor cells can be achieved through the pathway of Fas-FasL axis. Early growth response 3 (EGR3), as a novel zinc finger transcription factor, is a newly discovered tumor suppressor gene, which can inhibit the growth of hepatocellular carcinoma, gastric cancer, and other cancer cells by up-regulation of Fas ligand [77]. Zhang et al. [78] found that the low levels of EGR3 were significantly discovered in hepatocellular carcinoma tissues and various hepatoma cell lines (PLC/PRF/5, HCC-LM3, Huh7, and HepG2), but could be explored in human normal hepatic cell lines (L02). The experimental results of nude mouse models showed that the expression of FasL in xenograft tumor tissues with high expression of EGR3 was also significantly increased. Both EGR3 overexpression plasmid and FasL siRNA were co-transfected into hepatocellular carcinoma cells to silence the FasL gene obviously, which could hinder the anti-proliferation and pro-apoptotic effects. It was suggested that EGR3 could enhance the inhibition against hepatoma cells by up-regulation of FasL. NK-like (NKL) cells and isolated human peripheral blood NK cells were respectively transfected with chemically synthesized RNA-30C analogues and RNA-30C inhibitors in the research of Ma et al. [79]. Flow cytometric analysis revealed that the exogenous miR-30c mimics could effectively enhance the expression of membrane NKG2D and CD107a on the surface of NKL cells, and also could significantly enhance the cytotoxicity of NKL cells against SMMC-7721 cells via up-regulation of NKG2D, and also could trigger the high levels of FasL expression on both NKL cells and NK cells from peripheral blood. The signal transduction of FasL-associated death domain (FADD) via the apoptosis cascade could ultimately lead to apoptosis of SMMC-7721 tumor cells.

Adriamycin (ARG) has been currently recognized as a multifunctional chemotherapeutic drug with anti-inflammatory, anti-viral, anti-cancer, and other functions [80]. The data from Lu et al. [81] showed that apoptosis in HepG2 cells and Smmc7721 cells could be induced by Adriamycin via activation of the Caspase-8 signaling pathway, up-regulation of Fas/FasL expression, and increasing the secretion of TNF-alpha. The apoptotic effect of the former was significantly stronger than that of the latter. This indicated that Fas/FasL-related signal transduction pathways might play an important role in the anti-cancer process. Oncolytic viruses (OVs) have double functions of direct cytotoxicity and induction of effective anti-tumor immune responses [82]. The research reports from Chen et al. [83] showed that the intratumoral injection of measles virus vaccine strain Edmonston (MV-EDM) could induce the expression of specific ligand MICA and MICB on the surface of hepatoma cells, and thereby could significantly enhance the cytotoxicity of CD8^+^NKG2D^+^ T cells to hepatocellular carcinoma. It could also enhance the expression of FasL in CD8^+^NKG2D^+^ cells, but had no effect on the expression of Fas in hepatoma cells. These results suggested that the NK cells with activated NKG2D receptors could overexpress FasL, and activate Fas-mediated apoptotic signals, and then remarkably enhance its anti-tumor effects.

### 5.4. Perforin Secreted by NK Cells Exerts Anti-hepatocarcinoma Activity by Activating NKG2D

Perforin, also known as C9-related protein or cytolysin, is a glycoprotein with a molecular weight of 67 kDa, which exists in cytotoxic granules of NK cells and cytotoxic T lymphocytes. The mature perforin molecule is composed of 534 amino acid residues with a molecular weight of 56–75 kDa and an isoelectric point (IP) of 6.4 [84]. Li et al. [85] observed the differential expression of NK cells in tumorous tissue infiltrating lymphocytes (TILs) and non-tumorous tissue infiltrating lymphocytes (NILs) and PBMCs of patients with HCC by multicolor fluorescence activated cell classification analysis. It was found that NK cells in TILs and NILs were only 6.324 ± 1.559 and 14.52 ± 2.336, respectively. The abundance level of NK cells was significantly reduced and three other types of cells with positive Foxp3+ were explored in local tumorous tissues, which resulted in the decrease of IFN-γ and perforin secretion. The abundance of NK cells decreased significantly in local tumors, but three other types of cells with positive Foxp3+ were found, which resulted in the decrease of IFN-γ and perforin secretion. However, no similar results were observed in PBMCs of patients with hepatocellular carcinoma. The research had suggested that NK cell phenotypes were differentially expressed in local tumor tissues and peripheral blood of patients with hepatocellular carcinoma, and hypofunctional NK cells in local cancer lesions were difficult at playing an anti-cancer role.

The NK cells of CD56^+^CD16^+^ subtype account for about 90%–95% of the total number of NK cells in peripheral blood, and have high cytotoxic activity by secreting high levels of cytotoxic granular proteins, such as perforin. The correlation between the serum granulin–epithelin precursor (GEP) and NK cell activity in patients with HCC in the data of Cheung et al. [86] had been demonstrated that the level of GEP in HCC tissues was significantly higher than that in non-tumor liver tissues, and was not related to the number of NK cells in the peripheral blood of patients with HCC. However, it was negatively correlated with the expression of NKG2D and CD 69 on the surface of NK cells. The immunohistochemical staining showed that the expression of GEP was in a type of high and low in HCC tissues, and the number of NK cell infiltration was significantly decreased in the high GEP expression group. It was noteworthy that perforin production and cytotoxicity of NK cells returned to the same level as that of healthy NK cells after GEP inhibition in HCC cells. It was suggested that inhibition of GEP in hepatocellular carcinoma cells could prevent further damage of NK cell function in patients. When the GEP monoclonal antibody A23 was co-cultured with hepatocellular carcinoma cells in vitro, NK cells could be reactivated, the production of GEP and soluble ligand sMICA production were all reduced, so that the expression NKG2D was up-regulated, IFN-gamma and perforin production were significantly increased, and the cytotoxic effect was ultimately increased.

The latest study of 236 patients with liver cancer came from Sun et al. [87] had shown that CD96^+^NK cells failed to secrete enough interferon gamma (IFN-γ) and tumor necrosis factor alpha (TNF-α). The expression levels of perforin 1 (PRF1) and granzyme B (GZMB) were significantly decreased, which was positively correlated with the deterioration of the disease and negatively correlated with disease-free survival (DFS) and overall survival. Blocking the interaction of CD96-CD155 in vitro could specifically increase the cytotoxicity of NK cells on HepG2 cells. These results had been suggested that anti-hepatocellular carcinoma could be achieved by inhibiting the activity of CD96 molecule of NK cells and promoting the secretion of IFN-γ, TNF-α, PRF1, and GZMB from NK cells.

### 5.5. NKG2D Signaling Induces High Expression of CD16 on NK Cells Exerts Anti-hepatocarcinoma Activity

CD16, also known as Fc gamma RIII(FcγRIII), is a type III gamma receptor and a group of differentiated antigens expressed on the surface of NK cells, neutrophils, monocytes, and macrophages [88]. NK cells are congenital lymphocytes that mediate cytotoxic responses to viral infection and/or tumor cells. CD16 is a specific molecule expressed on the surface of NK cells and an important marker for identification to NK cells [89]. In humans, CD16 exists in the form of Fc gamma RIIIa (CD16a) and Fc gamma RIIIb (CD16b), and they have 96% homology in the extracellular immunoglobulin binding region. Most peripheral blood NK cells are CD56^+^CD16^+^ effector cells, only a small part of which belongs to CD56^+^CD16^−^ cells. Their function is regulated by a delicate balance between inhibitory and activating receptors, in which the CD16 low affinity receptor binds to the Fc end of IgG1 and mediates NK cells to recognize target cells through the Fab end of IgG1, so that the strong cytotoxic effects can be achieved [90]. This phenomenon is also called antibody-dependent cellular cytotoxicity (ADCC). The recent studies have found that the shedding of CD16 facilitates the formation of immune synapses in NK cells, enhances NK cells motility, and promotes the binding of CD16 to the target cells. Srpan et al. [91] observed retroviral transduced NK92/CD16 cells under a microscope. It was found that the repetitive activation of the Fc type III receptor (CD16) could reduce the secretion of perforin in an individual NK cell. Repeated stimulation of NKG2D could also reduce the secretion of perforin single NK cell, but the single stimulation of CD16 could not rescue the situation. Activation of CD16 could trigger the assembly of lysokinase, while inhibition of the shedding of CD16 was not conducive to the separation of NK cells from target cells. It had been suggested that the shedding of CD16 not only contributed to the formation of immune synapses and cell survival of NK cells, but also played a positive immune response to target cells.

Lee et al. [92] successfully amplified a large number of the cytotoxic natural killer cells by the use of radiation irradiation of autologous peripheral blood mononuclear cells and induction of anti-CD16 monoclonal antibody. It was found that this method could effectively amplify NK cells and obtain high purity and activating NK cells with less T cell contamination. The amplified NK cell surface activating receptor CD107A was significantly up-regulated and could secrete more IFN-γ, and had highly cytotoxicity to many kinds of cancer cells in vitro and in vivo. Subsequently, Chen et al. [93] explored the effects of CD16 overexpression in NK cells induced by monoclonal antibodies against the tumor embryo protein GPC3 (glypican-3) target to HCC. The results of immunohistochemistry (IHC) showed that GPC3 and CD16 could be expressed on tumor cells and NK cells of peripheral blood, respectively. The codrituzumab targeted drugs could successfully induce high expression of GPC3 and CD16, which was beneficial to improve the condition of patients. If the levels of GPC3 and/or CD16 decreased, the curative effects would be reduced. It had demonstrated that GPC3 and CD16 could be used as a novel compound biomarker to evaluate the curative effect of patients with liver cancer. The activating NKG2D could induce NK cells to express CD16 at a high level and further enhance the targeted cytotoxicity against cancer [94]. It was noteworthy that the fusion protein of NKG2G/NKG2DL had two-way regulatory functions [95]. On the one hand, it could directly up-regulate the expression of NKG2DL on the surface of hepatoma cells, to be recognized by NK cells. On the other hand, the up-regulation of NKG2D on the surface of NK cells also might significantly enhance the ADCC, degranulation, and other biological effects of NK cells, and played a crucial role in inhibiting the proliferation of hepatoma cells.

### 5.6. TRAIL Expressed on NK Cells Exerts Anti-hepatocarcinoma Activity by Activating NKG2D

The tumor necrosis factor-related apoptosis inducing ligand (TRAIL), also known as the apolipoprotein 2 ligand (APO2L), is a type II membrane protein with relative molecular mass of 32.5 kDa, containing three functional parts of the cytoplasm region (14aa), transmembrane region (26aa), and extracellular domain (241aa), which belong to the member of the tumor necrosis factor superfamily [96]. Its carboxyl terminal is located in the extracellular domain with a typical beta sandwich structure formed by several beta sheets, which are regarded as the major functional group with highly conserved. The amino terminal is located in the extracellular domain without a signal peptide sequence. TRAIL is specifically bound to the related receptor through a subunit structure composed of homologous trimers in the C terminal of the extracellular domain. The multiple tumor cells and transformed cells can be induced to apoptosis and inhibit through the pathway of caspase, death receptor, and mitochondria-related apoptosis [97]. It is exciting that the apoptosis induced by TRAIL in normal tissue cells of the body have not been confirmed.

As early as 2012, Ohira et al. [98] were surprised to find that liver mononuclear cells (LMNCs) extracted from perfusion fluid of deceased donor liver transplantation contained a higher percentage of NK cells than those of PBMCs from the same donor. Under stimulation by IL-2, NK cells were activated and the level of TRAIL was significantly increased. Compared with PBMCs, IL-2-stimulated LMNCs had stronger cytotoxicity to K562 target cells (*P* < 0.01), and could further reduce the risk of graft-versus-host disease (GVHD). These results suggested that the transfer of IL-2-activated NK cells from the perfusion fluid of deceased donor liver transplantation could be a novel treatment for hepatocellular carcinoma via the pathway of up-regulating the expression of TRAIL. On the basis of the successful construction of human interleukin-15 gene-modified NKL cells (NKL-IL15) in the research report of Jiang et al. [99] the female BALB/c xenograft nude mice (six weeks old) were used as an experimental animal model to confirm its quite strong inhibitive action to the growth of transplanted human hepatocellular carcinoma. The cytotoxicity effect of NK cells was measured by MTT, and the expression levels of TRAIL, TNF-α, IFN-γ, FasL, CD107a, perforin, and granzyme B from NKL cells were detected by the ELISA method. The results showed that NKL-IL15 cells could secrete more granular enzymes B, IFN-γ, TNF-α, and others, through the pathway of high expression of NKP80 and TRAIL, which had stronger cytotoxicity against HepG-2 cells than ordinary NKL cells. On the other hand, the secreted IFN-gamma and TNF-alpha could further induce the high expression of NKG2D ligand in hepatoma cells so as to enhance the sensitivity of NKL-IL15 cells to target cells. It had been suggested that IL-15 could significantly enhance the antitumor effects of NK cells through a multi-channel [100].

### 5.7. Important Issues Concerning the Role of NKG2D in the Immune Response against Hepatocellular Carcinoma

NK cells can be enriched in the microenvironment of the liver. The expression of NKG2D, an active receptor, plays a critical role in the innate immunity of NK cells against the malignant transformation of hepatocellular carcinoma [101]. The data of Cai et al. [102] showed that the number of CD56^dim^CD16^+^ NK subsets displayed a dramatic reduction in peripheral blood of HCC patients, and also demonstrated a significant reduction in local tumor regions than that in nontumor areas. Both these peripheral and tumor-infiltrating NK cells exhibited the poorer capacity to produce a cytotoxic ability and IFN-γ production. It is worth noting that hepatoma cells also can escape the innate immunity of NK cells mediated by NKG2D via the way of down-regulation of NKG2DL. Pollicino et al. [103] showed that HBV could significantly inhibit the expression of MICA in the HepG2.2.15 hepatoma cell line. However, by inhibiting HBV replication in HCC cells in transgenic mice, the expression of MICA in HCC cells could be restored, and NK cells could be induced to play a cytotoxic effect on the target cells. Consequently, a type of tumor-specific expression pattern of HBV might have existed in HCC, and NK cells could specifically recognize HCC cells through the MICA-NKG2D axis.

### 5.8. Targeted Stimulation of NKG2D Improves the Therapeutic Effect of NK Cells on Hepatocellular Carcinoma Immunotherapy

Targeted stimulation of NKG2D is a novel strategy to improve the immunotherapeutic effects of NK cells on patients with hepatocellular carcinoma. Kamiya et al. [104] used K562-mb15-41BBL cell line as a stimulant to obtain a large number of NK cells activated in the peripheral blood of healthy adult volunteers in vitro. The high expression of NKG2D-CD3ζ-DAP10 could significantly enhance NK-cell cytotoxicity against the HCC line. Therefore, when the same donor was injected with NKG2D modified NK cells, the tumor growth was significantly reduced and the overall survival rate was significantly improved. The data had demonstrated that the activated and expanded NK cells after genetic modification with NKG2D-CD3ζ-DAP10 could effectively kill HCC cells. Recently, Han et al. [105] successfully constructed a novel antibody-ligand fusion rG7S-MICA (also known as bi-specific antibody, BsAb), which was composed of an anti-CD24 single-chain variable fragment (scFv) and extracellular domains of MICA. The rG7S-MICA had a high affinity with the antibody and could effectively enrich NK cells to the tumor-bearing site of liver cancer in nude mice, and could enhance the anti-tumor activity by promoting the release of IFN-γ and TNF-α from NK cells via the MICA-NKG2D/Fc-FcR pathway. The most exciting feature was that it could also significantly enhance the anti-tumor effect of Sorafenib in vivo. Therefore, up-regulation of the level of NKG2D on the surface of NK cells in the peripheral blood and tumor tissues of patients with hepatocellular carcinoma could effectively improve the prognosis and prolong the overall survival rate of patients.

Another recent study of NKG2D-based CAR-T cells from Sun et al. [106] showed that the NK group 2 member D ligands (NKG2DLs), such as MICA or ULBP2 were overexpressed in hepatoma cell lines SMMC-7721 and MHCC97H. The CAR-T cells with extracellular domains containing human NKG2D, 4-1BB, and CD3 ζ signal domain (BBZ) had effective kill effects on hepatoma cell lines SMMC-7721 and MHCC97H cell lines with high expression of NKG2DL in vitro and in vivo, but had ineffective cytotoxic effects on SMMC-7721 or Hep3B cell lines with negative NKG2DL. This will provide a novel and effective treatment for HCC patients with positive expression of NKG2DL in the future.

An amazing and novel authoritative study from Sheppard [107] and others had been shown that NKG2D could unexpectedly promote the growth of hepatoma cells in a model of hepatocellular carcinoma. The growth of the tumor was compared in great detail both in NKG2D-deficient mice and NKG2D-sufficient mice. The memory CD8^+^T cells increased recruitment to the liver cancer lesions and increased the inflammatory reaction in the local microenvironment, which was beneficial to the malignant proliferation of the hepatocytes and promoted tumorigenesis. The growth of hepatoma cells could be achieved through an inflammation-cancer transformation pathway. This suggested that there might be another unusual molecular mechanism of the NKG2D/NKG2D ligand signaling pathway in the development of hepatocellular carcinoma, which needed further study in the future.

## 6. Conclusions

In summary, NK cells can be enriched in the human liver and have the potent antitumor ability through overexpression of NKG2D. NKG2D is the most important activating receptor on the NK cell surface with quite a complex genetic structure and multiple protein functional sites. The specific binding of NKG2D to the high expression of MICA, MICB, and ULBP on the surface of hepatoma cells can trigger multiple signaling pathways, such as phosphatidylinositol 3-hydroxy kinase (PI3K), phospholipase C Gamma 2 (PLCã2), c-Jun-NH(2)-terminal kinase (JNK), and others, which also further promote the anti-tumor effects of NK cells by secreting cytokines, enhancing ADCC effects, and initiates the apoptosis process [108]. NKG2D ligands can be expressed individually and collectively on the surface of liver cancer cells. However, the affinity of different ligands to NKG2D on the surface of NK cells is significantly different. How to realize the preferential combination of NKG2D and high-affinity ligands and precisely enhance the sensitivity of NK cells to hepatoma cells will become the focus of research and attention in the future.

## Figures and Tables

**Table 1:** The characteristics of activated and inhibitory receptors of NK cells.

Functional Classification	Structural Classification	Receptor in Human	Chromosome Localization	Number of Exons	Receptor in Mouse	Chromosome Localization	Number of Exons
Activating receptor	NCR	NKp46 or Ly94 or Mar-1	19p13.42	9	NKp46 or NCR1	7; 7 A1	7
		NKp30	6p21.32	5	\	\	\
		NKp44	6p21.1	6	\	\	\
		FcγRⅢ/CD16/FCG3/FCGR3	1q23.3	7	FcγRⅢ/CD16	1H3; 178.8 cM	9
	Type C lectin like receptor (CD94/NKG2 family)	NKG2D	12p12.3-p13.1	8\9\10	NKG2D (Main)	6 F3; 6 63.44 cM	9
		CD94/NKG2C	12p13.2	6	CD94/NKG2C	6; 6 F3	7
		CD94/NKG2E	12p13.2	7	CD94/NKG2E	6; 6 F3	7
		CD94/NKG2F	12p13.2	4	CD94/NKG2F	\	\
		CD161	12p13.31	6	CD161	6 F3; 6 63.09 cM	6
					Ly49d Ly49h	6 F3; 6 63.44 cM 6 F3; 6 63.44 cM	7 8
	KIRs	KIR2DS1	19q13.4	11	—	—	—
		KIR2DS4	19q13.42	8	—	—	—
		KIR2DL4	19q13.42	8	—	—	—
Inhibitory receptor	Type C lectin like receptor	CD94/NKG2A	12p13.2	9	Ly49a、c、g、i	6 F3; 6 63.44 cM 6 F3; 6 63.44 cM 6 F3; 6 63.44 cM 6 F3; 6 63.44 cM	9 7 9 9
		CD94/NKG2B	12p13.2	13	—	—	—
		PD-1 [26]	2q37.35	6	PD-1/Pdc1/Ly101	1;1D	5
		Siglec-7	19q13.41	7	—	—	—
	KIRs	KIR2DL1	19q13.42	11	—	—	—
		KIR2DL2 KIR2DL3	19q13.4 19q13.42	9 8	—	—	—
		KIR3DL1	19q13.42	9	—	—	—

**Table 2:** The differential expression of NKG2D in humans and mice.

Type	Human	Murine Mouse
NK cells	100%; Unactivated: 100%	100%
CD8^+^ αβT cells	After activation: 100%; Memory cells: 100%	After activation: 100%; Memory cells: 100%
CD4^+^ αβ T cells	Usually lack	Few express
γδT cells^†^	Peripheral γδT cells: 100%; Small intestinal epithelial cells: Absent	Dendritic epithelium γδT cells: 100%; Unactivated: absent. The spleen γδT Cells: 25%. Small intestinal epithelial cells: absent
NK1.1^+^ T cells	Undiscover	70% Positive
Invariant NKT cells (iNKT) [38]	Usually express	Usually express
Innate lymphoid cells (ILCs) [39]	Usually express	Usually express
Macrophages	Absent	LPS; IFNα/β or IFN-γ; After activation: 100%
Thymocyte	Single positive CD8+ T cells	Undiscover

^†^: According to the type of TCR, T cells can be divided into αβ+ T cells and γδ+ T cells. On the basis of the expression of the delta chain of TCR on the surface of γδ T cells, it can be further divided into four subgroups: Vδ1γδ T cells, Vδ2γδT cells, Vδ3γδ T cells, and Vδ5γδT cells [40]. Furthermore, in the light of the different functions of γδ T cells, they can be divided into regulatory γδ T cells (γδ Treg cells) [41,42], IL-17 producing γδ T cells (γδ T17 cells) [43], IFN-γ producing γδ T cells (IFN-γ+ γδ T cells) [44], human MutS homologue 2 (hMSH2) specific γδ T cells [45], and IL-6 secreting γδ T cells [46].
